# Slings in iatrogenic male incontinence: Current status

**DOI:** 10.4103/0970-1591.65423

**Published:** 2010

**Authors:** Fabrizio Gallo, M. Schenone, C. Giberti

**Affiliations:** Division of Urology, Department of Surgery, San Paolo Hospital, Savona, Italy

**Keywords:** Iatrogenic disease, male, suburethral sling, urinary incontinence, urologic surgical procedure

## Abstract

**Objectives:**

The increasing number of prostatectomies entails an increasing number of patients suffering from iatrogenic incontinence despite improved surgical techniques. The severity of this problem often requires invasive treatments such as periurethral injection of bulking agents, artificial urinary sphincter (AUS) implantation, and sub-urethral sling positioning. The artificial urethral sphincter has represented, until today, the gold standard but, in the recent years, sling systems have been investigated as minimally invasive alternative options. Today, three different sling procedures are commonly performed: bone-anchored, readjustable, and trans-obturator slings systems. The aim of this review is to critically report the current status of sling systems in the treatment of iatrogenic male incontinence.

**Materials and Methods:**

MEDLINE and PubMed databases were searched and all articles between 1974 and 2009 were evaluated.

**Results:**

With regard to bone-anchored, readjustable, and trans-obturator slings systems, cure rates ranged between 58.0% and 86.0%, 55.5% and 73.0%, and 40.0% and 63.0%, respectively, while major complication rates ranged between 0 and 14.5%, 10.0 and 22.2%, and 0 and 10.0%, respectively.

**Conclusions:**

Suburethral slings are the only alternative techniques which can be favorably compared with the AUS, showing more advantages with respect to AUS implantations which are mainly represented by a quick and less invasive approach, low morbidity, and low costs. In spite of the difficulty in identifying the most effective sling procedure, overall, sling systems can be recommended for patients with persistent mild or moderate incontinence. However, the indication can also be extended to patients with severe incontinence, after appropriate counseling, allowing AUS implantation in the event of sling failure.

## INTRODUCTION

Male stress urinary incontinence (SUI) affects a substantial number of patients after radical or simple, open or transurethral, prostatectomy.[1–2] Its frequency varies dramatically, ranging from 2.5% to 67%, and this broad range of incontinence rates has been attributed to differences in continence assessing methodology, surgeon experience, and patient selection.[3–4] Nevertheless, iatrogenic incontinence remains a critical determinant in postoperative health-related quality of life.[5]

Initial management of SUI is usually conservative and includes the use of diapers or pads. In the early postoperative period, mild degrees of incontinence may be improved by pelvic floor muscle training, biofeedback, or electrical stimulation which could help to cure the patients within the first 6-12 months after surgery.[6–7] Surgical treatments are therefore generally not recommended within this time interval. No pharmacologic treatment has shown to be effective in randomized, controlled trials. However, anticholinergic drugs can be used in cases of mixed incontinence to cure the urgency, while duloxetine seemed to improve symptoms when it was used off label.[8–9]

When conservative and pharmacologic managements fail, patients are usually offered one of the following invasive alternatives: balloon compression device, injections of bulking agents, artificial urinary sphincter (AUS) placement, and sling positioning. The use of urethral or bladder neck balloon compression devices have been reported by a few groups showing a short-term cure rate of 60-70%.[10–11] Transurethral injections of various bulking agents have been used for decades due to its minimal invasivity, safety, and good tolerability. However, effectiveness is usually temporary, requiring multiple injections and long-term results have been disappointing so far, with cure rates reaching only reaching 20-40%.[12–13] The AUS is the best long-term surgical treatment with consistently high patient-satisfaction rates (75-94%) and it has represented, until today, the gold standard by which other surgical management must be compared.[14–15] Despite its remarkable results, the AUS is an expensive mechanical device that requires manual dexterity and an adequate mental capacity to be used. In addition, a revision rate of more than 20% at 5 years was reported due to mechanical failure, infection or cuff erosion, and spurring interest in alternative surgical procedures.[16]

Sling procedures are conceptually attractive because they are inexpensive, not mechanical, and allow for physiologic voiding without significant obstruction.[17–18] The use of fixed urethral compression for the treatment of male SUI began in 1961 when Berry used acrylic prostheses to compress the ventral urethra against the urogenital diaphragm, followed by the different sling procedures developed by Kaufman and Hauri in the 1970s.[19–23] Since then, various techniques of bulbar urethra compression using biologic or synthetic materials have been described, showing good success rates, comparable favorably with those obtained after placement of an AUS, although no randomized studies concerning these two procedures have been reported in the literature.[24–27]

Today, three different sling procedures are commonly used to treat iatrogenic male SUI: bone-anchored sling, readjustable sling, and trans-obturator sling systems.[28–30]

The aim of this review is to critically report the current status of sling systems in the treatment of iatrogenic male incontinence.

## MATERIALS AND METHODS

We searched MEDLINE and PubMed for original articles published between 1974 and 2009 using the terms “postoperative male incontinence” and “suburethral sling.” Current publications and data most relevant to urologists were examined.

## RESULTS

### Bone-anchored sling systems

The bone-anchored sub-urethral sling (BAUS) implantation foresees, via a perineal incision, the positioning of a mesh under the bulbar urethra, attached to both ischiopubic rami by two-three titanium screws. The sling can be made of biologic, synthetic, and mixed (biologic and synthetic) materials.

The first data concerning this procedure were reported in 2001, when, in a cohort of 16 patients, a short-term continence rate of 86% with no complications was reported.[28] In the following years, these good results were revised based on bigger enrollments and longer follow-ups[31–34] showing a continence rate ranging between 58% and 67% [Table 1].

**Table 1:** Outcomes from the main articles concerning bone-anchored sling systems

Author	No. PTS	F.U. (months)	Cured PTS (%)	Improved PTS (%)	Failed PTS (%)	Complication rate (%)
Madjar *et al*[28]	14	12	86.0	14.0	0	0
Ullrich *et al*[31]	36	25	67.0	14.0	19.0	0
Comiter *et al*[32]	48	48	65.0	15.0	6.0	7.0
Fischer *et al*[33]	62	15	58.0	-	42.0	14.5
Giberti *et al*[34]	42	41	62.0	8.0	12.0	5.0

No. PTS = number of patients; F.U = follow-up

Overall, all the authors reported a similar low rate of minor complications (0-14.5%), mainly represented by postoperative perineal pain, urge incontinence, transient urinary retention, and sling infection which were generally treated with drugs and did not require sling removal or reoperation.[27,30–33] Only Fischer *et al*. reported a case of infection with urethral erosion.[33]

Concerning the sling materials, better long-term results have been reported using synthetic sling (silicone-coated), due to its higher tensile strength.[34–36]

As regards preoperative factors which could influence the surgical outcomes, lower success rates were generally reported in patients with severe SUI, history of anti-incontinence procedures, and radiotherapy.[28,31–34] In particular, radiotherapy resulted the main predictor of failure and sling infection, as reported in the papers by Fassi-Fehri *et al*. and Giberti *et al*, who showed a failure rate of 85% and 75% respectively in case of a past history of pelvic radiotherapy.[36–37] The good results reported by Giberti *et al*. in severe SUI would suggest the use of BAUS also in these patients before the implantation of more invasive and expensive dispositive as the AUS.[34–37]

In the event of suboptimal continence following sling surgery, the subsequent implantation of an AUS still remains a valid option with equally results comparing to patients with no surgical pre-treatment.[38]

### Readjustable sling systems

The male readjustable systems (MRSs) are suburethral sling devices which permit an effective regulation of the sling tension not only during surgery but also in the first postoperative days and at any time during the patient’s life. This possibility of suburethral pressure control should represent the main advantage of this procedure in order to cure incontinence avoiding urinary retention.

The use of two different MRSs have been reported in the literature[29,39–40] [Table 2].

**Table 2:** Outcomes from the main articles concerning readjustable sling systems. No. PTS = number of patients; F.U = follow-up

Author	No. PTS	F.U. (months)	Cured PTS (%)	Improved PTS (%)	Failed PTS (%)	Complication rate (%)
Sousa-Escandon *et al*[39]	51	32	64.7	19.6	15.7	15.8
Campos-Fernandes *et al*[40]	18	26.3	55.5	11.1	33.4	22.2
Romano *et al*[42]	48	7.5	73.0	10.0	17.0	10.0

The REMEEX system is composed of a synthetic monofilament sling, connected via two monofilament traction threads to a suprapubic mechanical regulator. The regulator is a permanent subcutaneous implant over the abdominal rectum fascia 2 cm above the pubis which can be attached to an external manipulator in order to adjust sling tension. The first results for this system were published in 2004 by Sousa-Escandon *et al*, who reported success in five out of six patients (83%) treated.[29] In a multicentre European study with 51 patients with a mean follow-up of 32 months, 33 patients (64.7%) were cured, 10 (19.6%) improved while 8 (15.7%) failed. As regards major complications, the system was removed in three cases due to urethral erosion and infection in one (2%) and two (4%) patients, respectively.[39] Bladder perforation was reported in five cases (9.8%). Among minor complications, most patients felt perineal discomfort or pain, which was due to a mild perineal hematoma in three cases and was easily treated with oral drugs. Satisfactory results were lower in patients with severe incontinence or a previous history of pelvic radiotherapy. Similar results were reported by Campos-Fernandes *et al*. in 18 patients.[40]

The ARGUS system is another adjustable suburethral sling procedure composed of a radiopaque cushioned system with silicone foam for soft bulbar urethral compression, two silicone columns formed by multiple conical elements, which are attached to the pad and allow system readjustment, and two radiopaque silicone washers which allow regulation of the sling tension. This procedure was first described by Sierra *et al*.[41] In a cohort of 48 patients with a mean follow-up of 7.5 months Romano *et al*, showed a cure rate of 73%. As concerns major complications, the sling was removed in five cases due to urethral erosion and infection in three (6%) and two (4%) patients, respectively.[42]

### Trans-obturator sling systems

Following the success of this approach in curing women’s SUI with highly satisfactory results, placement of a trans-obturator sling has also been described as a new option to treat male postoperative SUI with a nonobstructive, functional approach.[30] Furthermore, the trans-obturator approach is expected to minimize the risk of bladder, bowel, and vessel injuries which can be observed during the blind, retropubic passage of a needle through the pelvic space, particularly after retropubic prostatectomy.

However, to our knowledge, there are very few available study results which show a remarkable number of patients and a mid-term follow up.

Two different surgical variants of this technique have been proposed [Table 3].[30,43]

**Table 3:** Outcomes from the main articles concerning trans-obturator sling systems. No. PTS = number of patients; F.U = follow-up

Author	No. PTS	F.U. (months)	Cured PTS (%)	Improved PTS (%)	Failed PTS (%)	Complication rate (%)
Rehder *et al*[30]	20	6	40.0	30.0	30.0	10.0
de Leval *et al*[43]	20	6	45.0	40.0	15.0	0
Gozzi *et al*[44]	67	6	52.0	38.0	10.0	-
Bauer *et al*[45]	124	12	51.4	25.7	22.9	0.8
Cornu *et al*[46]	102	13	63.0	17.0	20.0	0

The AdVance system is an outside-in trans-obturator sling. The first results were reported by Rehder *et al* and confirmed by Gozzi *et al*, who showed cure and improvement rates of 52% and 38% respectively with low morbidity after 6 months follow-up.[30–44] Bauer *et al* confirmed these results at 1 year of mean follow-up reporting cure and improvement rates of 51.4% and 25.7%, respectively. However, these outcomes were assessed only in patients with a detectable hypermobility of the sphincter region and good residual sphincter function due to the absence of intrinsic sphincter deficiency. As regards major complication, only one case of urethral erosion (0.8%) was reported in this series.[45]

Better results were reported by Cornu *et al* who conducted a prospective evaluation on 102 patients with a median follow-up of 13 months, showing cure and improvement rates of 63% and 17%, respectively. However, as reported by the authors, these better results should be considered with caution since they are related to patients with only mild or moderate incontinence excluding those patients with severe SUI and a higher risk of failure. Based on these aspects, the authors suggested assessing the degree of incontinence before proposing this treatment. Concerning major complications, no case of sling infection, urethral erosion, or reoperation were recorded.[46]

An inside-out trans-obturator sling has also been proposed by de Leval *et al*. The authors showed at 6 months mean follow-up, cure and improvement rates of 45% and 40%, respectively. No sling infection, persistent pain, bladder, urethra, bowel, or nerve complications were encountered.[43]

As reported previously for trans-obturator sling systems, strictures and radiation-related lesions of the urethra were suspected risk factors for a lower success rate due to the decreased tissue compliance.[30,43–46]

## CONCLUSIONS

In spite of the new surgical techniques, iatrogenic incontinence is still the most feared complication for men, especially following radical prostatectomy. The management of this bothersome problem is almost controversial due to the lack of conclusive data regarding the optimal timing to begin the treatment and the best option to choose. Generally, urologists proposed non-invasive therapies for early incontinence with surgical options reserved for case of persistent or severe incontinence.

Although there was a lack of prospective randomized studies concerning the different anti-incontinence surgical procedures, the AUS represented the best long-term treatment with consistently high patient-satisfaction rates (75-94%) and it was, until today, the gold standard by which other surgical management was compared.[14–15] However, technical problems related to the AUS management, the long-term complications, the expensive costs, and the increasing patient demand for minimally invasive treatment options have spurred interest in other surgical procedures in order to avoid AUS implantation.[16]

Suburethral slings are the only alternative technique which can be favorably compared with AUSs, showing further advantages with respect to AUS implantation. Firstly, the sling procedure is a quicker and less invasive procedure which can be easily performed by many more surgeons than AUS implantation. Secondly, the slings are physiologic devices of urethral compression which do not require mechanical manipulation and can consequently be proposed to all patients regardless of their mental capacity or manual dexterity. Thirdly, although the sling procedures reported lower cure rates than those showed after AUS implantation, these outcomes are durable and not limited by mechanical failures which can occur during follow-up of the AUS procedure. Fourthly, the sling compression of the ventral bulbar urethra over a relatively large surface, without incision of the bulbourethral muscle, can better respect urethral tissues than the AUS with its cuff encircling the entire circumference of the urethra on a smaller contact surface, minimizing the risk of urethral atrophy and subsequent erosion. In contrast, significant erosion rates, ranging from 5 to 15%, were reported after AUS placement. Fifthly, sling systems have significantly lower costs than AUS implantation.[14–17]

Furthermore, in the event of sling failure, the following AUS positioning can be a valid option with results comparing equally to those from patients with no surgical pre-treatment.

However, the crucial point of sling procedures remains the evaluation of the appropriate tension in order to produce a satisfactory continence, minimizing the risk of failure and complications. In fact, a low sling tension would not cure incontinence while an excessive tension could produce urinary retention, urge incontinence, or urethral erosion.

As regards the three sling techniques reported in the literature, identification of the most effective procedure is still very difficult due to the lack of comparative studies and the differences reported in follow-up. In this setting, the BAUS procedure represented the most tested technique with remarkable and long-lasting results.[28,31–34] The MRSs seemed to represent an interesting and useful innovation although further studies are needed to show their supposed better efficacy.[39–42] The role of the trans-obturator sling procedure must still be confirmed in the follow-up, especially in patients with severe incontinence or intrinsic sphincteric deficiency.[30,43–46]

Overall, sling systems can be recommended for patients with persistent mild or moderate incontinence with remarkable results and low morbidity. However, the indication can also be extended to patients with severe incontinence after appropriate counseling, allowing AUS implantation in the event of sling failure.
